# The glutamine synthetase gene family in *Populus*

**DOI:** 10.1186/1471-2229-11-119

**Published:** 2011-08-25

**Authors:** Vanessa Castro-Rodríguez, Angel García-Gutiérrez, Javier Canales, Concepción Avila, Edward G Kirby, Francisco M Cánovas

**Affiliations:** 1 Departamento de Biología Molecular y Bioquímica, Instituto Andaluz de Biotecnología, Universidad de Málaga, 29071-Málaga, Spain; 2 Department of Biological Sciences, Rutgers University, Newark, New Jersey 07102, USA

## Abstract

**Background:**

Glutamine synthetase (GS; EC: 6.3.1.2, L-glutamate: ammonia ligase ADP-forming) is a key enzyme in ammonium assimilation and metabolism of higher plants. The current work was undertaken to develop a more comprehensive understanding of molecular and biochemical features of *GS *gene family in poplar, and to characterize the developmental regulation of *GS *expression in various tissues and at various times during the poplar perennial growth.

**Results:**

The *GS *gene family consists of 8 different genes exhibiting all structural and regulatory elements consistent with their roles as functional genes. Our results indicate that the family members are organized in 4 groups of duplicated genes, 3 of which code for cytosolic GS isoforms (GS1) and 1 which codes for the choroplastic GS isoform (GS2). Our analysis shows that *Populus trichocarpa *is the first plant species in which it was observed the complete *GS *family duplicated. Detailed expression analyses have revealed specific spatial and seasonal patterns of *GS *expression in poplar. These data provide insights into the metabolic function of GS isoforms in poplar and pave the way for future functional studies.

**Conclusions:**

Our data suggest that *GS *duplicates could have been retained in order to increase the amount of enzyme in a particular cell type. This possibility could contribute to the homeostasis of nitrogen metabolism in functions associated to changes in glutamine-derived metabolic products. The presence of duplicated *GS *genes in poplar could also contribute to diversification of the enzymatic properties for a particular GS isoform through the assembly of GS polypeptides into homo oligomeric and/or hetero oligomeric holoenzymes in specific cell types.

## Background

Glutamine synthetase (GS; EC 6.3.1.2, L-glutamate: ammonia ligase ADP-forming) catalyzes the ATP-dependent addition of ammonium (NH_4_^+^) to the γ-carboxyl group of glutamate to produce glutamine and acts as the center for nitrogen flow in plants. Glutamate synthase (Fd-GOGAT, EC 1.4.7.1; NADH-GOGAT, EC 1.4.1.1) then catalyzes the conversion of glutamine and 2-oxoglutarate to produce two molecules of glutamate, one of which participates in further ammonium assimilation via GS while the other donates reduced nitrogen for all nitrogen-containing biomolecules [1]. The ammonium assimilated by GS in the production of glutamine can come from various sources, including direct uptake from the soil, reduction of nitrate and nitrite, photorespiration, deamination of phenylalanine catalyzed by phenylalanine ammonia-lyase, and the catabolic release of ammonium during the mobilization of vegetative storage proteins and during senescence.

Multiple nuclear encoded GS polypeptides are expressed in photosynthetic and non-photosynthetic tissues of higher plants and these polypeptides are assembled into oligomeric isoenzymes located either in the cytosol or in the chloroplast [2,3]. Recently it has been reported that plant GS holoenzyme has a decameric structure composed of two face-to face pentameric rings of subunits, with active sites formed between every two neighboring subunits within each ring [4,5]. Phylogenetic studies of nucleotide and amino acid sequences have shown that genes for chloroplastic and cytosolic GS in plants form two sister groups with a common ancestor which diverged by duplication before the split between angiosperms and gymnosperms [6].

In angiosperms there are two main isoforms of GS, cytosolic GS (GS1) and a chloroplastic GS (GS2). This suggests that there are several distinct pathways for glutamine production, both spatially and temporally. In developing leaves, glutamine is mainly produced in chloroplasts through the activity of the GS2 isoenzyme. The ammonium assimilated into glutamine in young leaves is produced by nitrate reduction and through photorespiration [7,8]. Alternatively, cytosolic GS1 primarily generates glutamine for intercellular nitrogen transport. The cytosolic enzyme assimilates ammonium taken up from the soil and released in the biosynthesis of phenylpropanoids and nitrogen remobilization [9-11]. Thus, *GS1 *genes are differentially expressed in roots and in vascular tissues. Molecular analysis of genomic *GS *sequences from a number of angiosperm species has shown that the cytosolic *GS1 *genes belong to a small multigene family, whereas, the chloroplastic GS2 is encoded by a single gene [9,10].

GS plays a fundamental role in growth and development of woody plants [11,12]. In poplar, this critical role for GS has been clearly demonstrated through studies of transgenic poplars that express ectopically the pine cytosolic GS. Transgenic poplars exhibit enhanced vegetative growth [13,14], enhanced resistance to drought stress at both ecophysiological and enzymatic and non-enzymatic antioxidant levels [15], and enhanced nitrogen use efficiency [16]. These results clearly lead to the conclusion that in poplar GS activity is a limiting factor in growth and development. The current work was undertaken to develop a more comprehensive understanding of molecular and biochemical features of *GS *gene family in poplar, to establish an understanding of the roles of specific members of the poplar *GS *gene family during development, and to characterize the developmental regulation of *GS *expression in various tissues and at various times during the poplar perennial growth.

## Results

### Identification and structural analysis of poplar *GS *genes

A search of the *Populus trichocarpa *whole genomic sequence at the JGI [17] allowed us to identify regions containing *GS *sequences. Eight sequences containing a complete ORF as well as the structural and regulatory elements for a functional gene were retained for further study. The poplar genome data base also contains 9 *GS *pseudogenes as well as an additional *GS *gene showing a high identity level to the *GS *genes in archaebacteria. The full-length cDNAs (FLcDNAs) of the 8 *GS *genes were analyzed and the characteristics of the polypeptides encoded by their ORFs were compared (Table 1). The results of all these bioinformatic analyses allowed the identification of 6 genes coding for a cytosolic GS iosenzyme (GS1) and 2 genes coding for a plastidic GS isoenzyme (GS2). Additionally, our analysis suggests that the *GS *gene family in poplar is organized in 4 groups of duplicated genes, *PtGS1.1*, *PtGS1.2*, *PtGS1.3 *and *PtGS2*. According to the original identification numbers at the JGI database, poplar *GS1 *genes were named *PtGS1.1-710678 *and *PtGS1.1-831163*, *PtGS1.2-716066 *and *PtGS1.2-819912*, *PtGS1.3-827781 *and *PtGS1.3-834185*. Following the same criteria, poplar *GS2 *genes were named *PtGS2-725763 *and *PtGS2-820914*. The genetic distance between the different *GS *genes was calculated considering the complete genomic sequence of the individual members of the gene family confirming the existence of the *GS *gene duplicates.

**Table 1:** List of *GS *gene sequences containing a complete open reading frame (ORF) in the genome of *Populus trichocarpa*

Gene	FL cDNA (bp)	ORF (amino acids)	MW (kDa)	pI	Name	Isoenzyme
estExt_Genewise1_v1.C_LG_X4165	1299	432	U: 47894.2 P: 42291.5	U: 6.48 P: 5.34	PtGS2-725763	PtGS2
	
estExt_fgenesh4_pg.C_LG_VIII1790	1299	432	U: 47746.9 P: 42172.2	U: 6.48 P: 5.33	PtGS2-820914	

estExt_fgenesh4_pm.C_LG_IV0266	1074	357	39355.4	5.52	PtGS1.1-831163	PtGS1.1
	
estExt_Genewise1_v1.C_LG_II2125	1077	358	39448.5	5.95	PtGS1.1-710678	

estExt_fgenesh4_pg.C_LG_VII0739	1071	356	38973.0	5.53	PtGS1.2-819912	PtGS1.2
	
estExt_Genewise1_v1.C_LG_V3325	1071	356	39057.0	5.14	PtGS1.2-716066	

estExt_fgenesh4_pm.C_LG_XII0003	1071	356	39092.0	5.86	PtGS1.3-834185	PtGS1.3
	
estExt_fgenesh4_pg.C_1220090	1071	356	39207.2	5.81	PtGS1.3-827781	

U: Unprocessed protein

P: Processed

**Figure 1:**
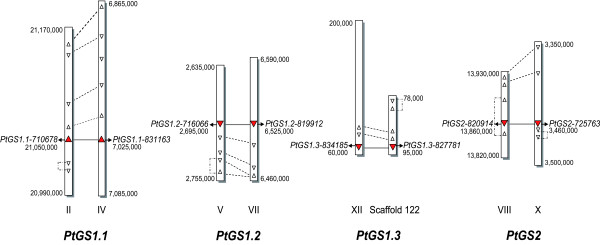
**Distribution of *GS *genes in the chromosomes of *Populus trichocarpa***. Linkage Groups (LG) numbers are indicated. PtGS1.3-827781 is located in the unassambled Scaffold 122. Arrows indicate the 5'-3' orientation of genes. Red arrows connected by horizontal solid lines are the duplicated *GS *genes. White arrows connected by dotted lines are duplicated collinear genes located adjacent to the positions where the *GS *genes are present. White arrows connected by dashed-dotted lines are internal duplicated genes. The position of genes is marked by the numbers of bp in each LG.

The four duplicated *GS *genes were positioned in the linkage groups (LG) or scaffolds present in the *Populus trichocarpa *genome (Figure 1). The genomic regions where the *GS *genes were located were examined in detail by determination of the open reading frames (ORFs) upstream and downstream of the specific *GS *genes and cross-alignment of these adjacent regions between the gene pairs. Several duplicated genes were collinearly positioned for the *PtGS1.1*, *PtGS1.2 *and *PtGS2 *duplicates. However, it was not possible to localize the *PtGS1.3 *duplicate because the region dowstream *PtGS1.3-827781 *was not present in the scaffold where the gene is located (Figure 1). Non-duplicated genes were also observed near the *GS *genes, as well as internal duplications located on the same chromosome.

**Figure 2:**
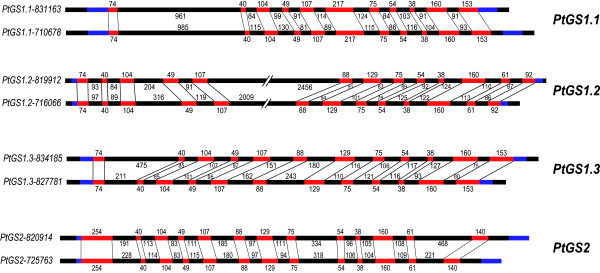
**The family of *GS *duplicate genes in *Populus trichocarpa***. Members of the family are represented as pairs of duplicated genes. The name of each pair is indicated on the right. Exons are in red, introns in black, and the UTR regions are in blue. The numbers of nucleotides are indicated for each exon and intron. Correspondence between segments is marked by vertical lines.

Structural analysis of the *GS *gene family in poplar was performed by comparison of the exon/intron organization. As shown in Figure 2 the size of the exons is generally well conserved in the four duplicates, *PtGS1.1*, *PtGS1.2*, *PtGS1.3 *and *PtGS2*. However, the genomic structure is substantially different at the intron regions with introns significantly divergent in size and sequence. In contrast to these observed differences among the gene duplications, the exon/intron boundaries are almost identical between the two members of each duplicate (Figure 2). The *PtGS2 *and *PtGS1.2 *duplicates contain 13 exons and 12 introns, the *PtGS1.3 *duplicate presents 12 exons and 11 introns, and the *PtGS.1.1 *duplicate contains 11 exons and 10 introns. Interestingly, exon 6 in the *PtGS1.1 *duplicate represents the fusion of exons 6 and 7 in the PtGS1.2, *PtGS1.3 *and *PtGS2 *duplicates. On the other hand, the last exon in the *PtGS1.1 *and *PtGS1.3 *duplicates represents the fusion of exons 12 and 13 in the *PtGS1.2 *duplicate. It is interesting to note the presence of an intron of more than 2 kb interrupting exons 5 and 6 in the *PtGS1.2 *duplicate.

### Comparative analysis of *GS *gene families in sequenced plant genomes

**Figure 3:**
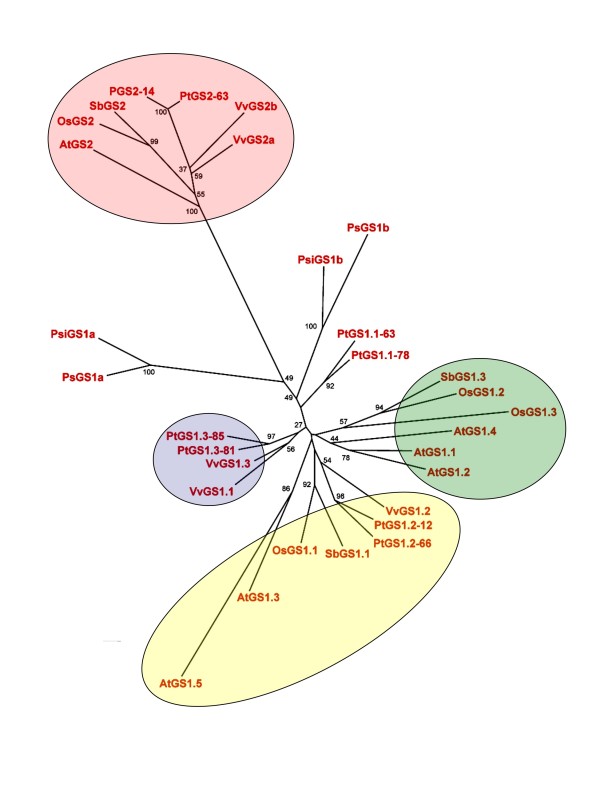
**Relationships between poplar and other *GS *gene families in plants**. Phylogenetic analyses of predicted full-length protein sequences were performed using the neighbor joining method. Tree was constructed as described. Pt: *Populus trichocarpa*. Os: *Oryza sativa*. Vv: *Vitis vinifera*. Sb: *Sorghum bicolor*. At: *Arabidopsis thaliana*. Ps: *Pinus sylvestris*. Psi: *Picea sitchensis*.

To examine the evolutionary relationships of poplar *GS *genes we performed a cladistic analysis based on deduced amino acid sequences, including the complete *GS *gene families from the sequenced genomes of Arabidopsis, rice, grape, sorghum and poplar. Pine and spruce *GS *genes were also included in this comparative analysis (Figure 3). Phylogenetic reconstruction at the molecular level shows the separation of cytosolic (GS1) and chloroplastic (GS2) sequences in angiosperms as two well differentiated clusters. Figure 3 also shows that poplar duplicates for *GS2 *and *GS1 *genes were distributed in the two clusters. *GS1 *genes from Arabidopsis, rice, grape and sorghum were distributed in three subfamilies and the *PtGS1.2 *and *PtGS1.3 *duplicates were clearly associated to two of these subfamilies. In contrast, the *PtGS1.1 *duplicate was outside the conserved GS1 subfamilies and was more closely aligned with the GS1 isoforms of gymnosperms that group outside the main subfamilies of GS1 in angiosperms. However, these data should be interpreted with caution because the support values of the clades are moderate.

### Regulatory regions in the poplar *GS *genes

**Figure 4:**
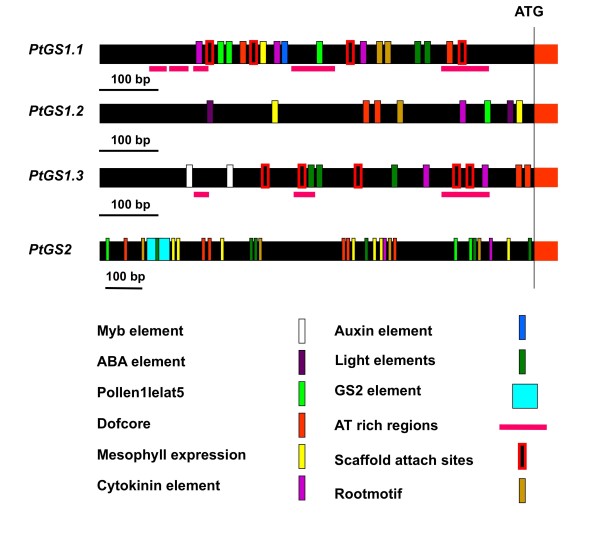
**The regulatory regions of the poplar *GS *genes**. The 5' upstream regions of GS genes are represented. Regulatory elements conserved in each pair of duplicated genes are marked in colours. The position of the ATG is marked on the right.

In order to get insight into the function of *GS *genes in poplar, the presence of regulatory elements in the 5'-upstream regions was investigated. According to results previously obtained in the structural and phylogenetic analyses, we decided to consider exclusively regulatory elements that were present in the two members of a *GS *duplicate (Figure 4). In the *PtGS1.1*, *PtGS1.2 *and *PtGS1.3 *genes, these common regulatory elements were found concentrated in the proximal region of the promoter (about 600 bp upstream the initiation of translation). In contrast, the presence of common regulatory elements spanned a major region in the promoter of the *PtGS2 *duplicate (about 1300 bp upstream the initiation of translation). Putative regulatory elements involved in the interaction with Myb trancription factors were identified exclusively in the *PtGS1.3 *duplicate. Light-responsive elements such as GATA boxes were identified in all gene duplicates except *PtGS1.2*. Regulatory elements involved in tissue-specific gene expression (mesophyll, roots) were identified in all genes except *PtGS1.3*, whereas ABA response elements were present in the promoters of *PtGS1.2 *duplicates. Boxes specific to cytokinin response were identified in all *GS *genes but auxin response elements were exclusively found in *PtGS1.1*. The poplar *GS2 *promoter contains a sequence of about 200 bp showing a 90% identity with light-regulatory elements that have been functionally characterized in the GS2 of pea and common bean [18]. Finally, the presence of AT-rich regions was detected in all GS promoters although they were much less abundant in the *PtGS2 *duplicate.

### Organ-specific expression of duplicate *GS *genes in poplar

**Figure 5:**
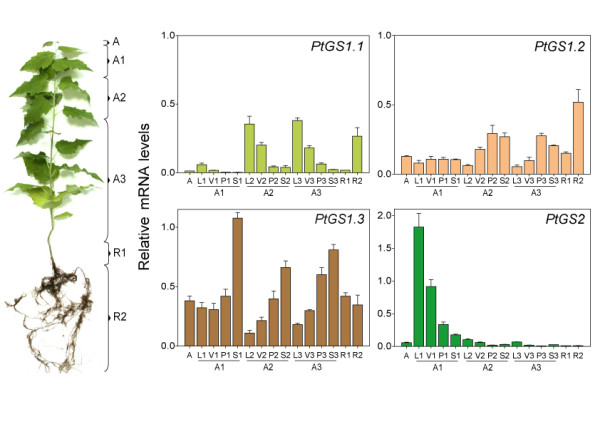
**Spatial distribution of *GS *gene expression in poplar trees**. Total RNA was extracted from different organs of 2-month-old hybrid poplar. A, meristematic apex. A1, A2 and A3, aerial sections from the top to the bottom of tree. L, leaf lamina. V, veins. P, petiole. S, stem. R1, primary root. R2, secondary root masses. Transcript levels of *PtGS1.1*, *PtGS1.2*, *PtGS1.3 *and *PtGS2 *were determined by real-time qPCR analysis as described. Expression levels are presented as relative values to reference genes (actin2 and ubiquitin). The histograms represent the mean values of three independent experiments with standard deviations.

To understand the regulation of the *GS *gene family in poplar and obtain further insight into the biological roles of members in the gene family, *GS *expression was precisely quantified spatial and temporally. Total RNA was extracted from different organs and the relative abundance of *GS *transcripts was determined quantitatively by real-time PCR (qPCR). In all cases the transcript levels were normalized by comparison with expression levels of reference genes (as described in Material and Methods). Two month-old hybrid poplars were divided into above-ground and root-regions (Figure 5). The aerial region included the meristematic apex (A), young leaves and stem internodes (A1), intermediate leaves and stem internodes (A2), mature leaves and stem internodes (A3). Aerial regions A1, A2 and A3 were further subdivided in lamina of the leaf (L), leaf vein (V) and stem (S). The root region included the main root close to the root crown (R1) and the secondary root masses (R2). As shown in Figure 5, gene expression profiles of *PtGS1.1*, *PtGS1.2*, *PtGS1.3 *and *PtGS2 *differed significantly in the samples examined. *PtGS1.1 *transcripts were particularly abundant in the aerial regions containing intermediate and mature leaves (A2 and A3) and in R2. Interestingly, maximum levels of *PtGS1.1 *expression were observed in the leaf lamina (L2, L3) with decreased abundance in the leaf veins (V2, V3). Minor levels of gene expression were observed in petioles (P2, P3) and stems (S2, S3). For the *PtGS1.2 *duplicate the highest transcript abundance was observed in the secondary root masses (R2), while about a half of this value was observed in petioles (P2, P3) and stems (S2, S3) of the aerial parts (A1 and A2). Much lower levels of *PtGS1.2 *transcripts were detected in remaining samples. Figure 5 also shows that expression of the *PtGS1.3 *duplicate was predominant among the poplar *GS1 *genes, and high levels of *PtGS1.3 *transcripts were observed in the apex, aerial and root sections. Furthermore, levels of *PtGS1.3 *transcripts were highest of the poplar *GS *gene family in the apex. It is important to note that in the aerial sections, expression of *PtGS1.3 *was clearly associated with samples enriched in vascular tissue, such as petioles (P1, P2 and P3) and stems (S1, S2 and S3) whereas lower levels of gene expression were observed in the leaf lamina in all sections examined. Finally, analysis of the *PtGS2 *duplicate revealed that the transcripts of this family member were the most abundant in the young leaves (A1), and decreased progressively from the top to the bottom of the tree, with the lowest values detected in the roots.

**Figure 6:**
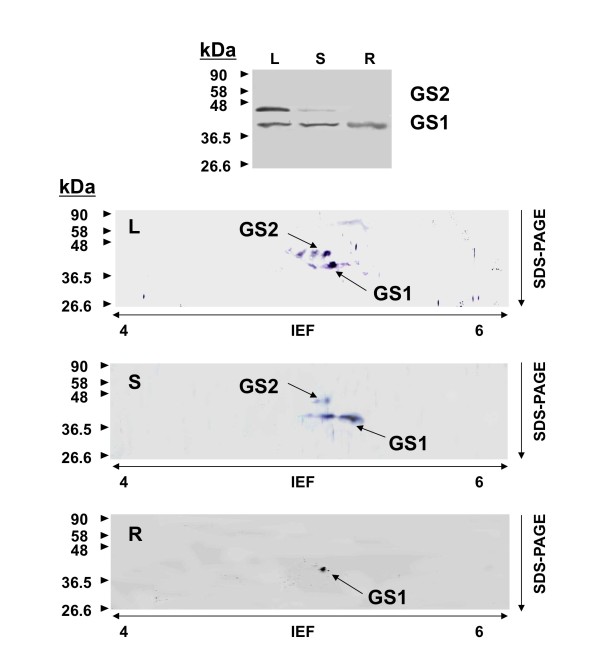
**Analysis of GS polypeptides in poplar trees**. Proteins were extracted from different organs of 2-month-old hybrid poplar. L, leaf. S, Stem. R, root. Thirty micrograms of proteins per lane were separated by PAGE and then transferred to a nitrocellulose membrane, where the proteins were probed using a specific antibody developed against pine GS [19]. A, One dimensional analysis. B, Two dimensional analysis. Spot variants in two dimensional gel separation of GS polypeptides has been previously reported [31] which could be the result of post-translational modifications. The molecular size (kDa) of protein markers are indicated on the left. Major GS spots observed in the different experiments are marked by arrows.

In order to determine if there was a correspondence between the expression patterns of the *GS *transcripts and the distribution of GS polypeptides, we examined the distribution of GS polypeptides in different organs. Total proteins were extracted from leaves, stems and roots of two month-old poplar trees and GS polypeptides in these organs were identified by western blot analysis using antibodies raised against pine GS [19]. It has been previously reported that these antibodies were able to recognize specifically poplar GS polypeptides [13]. Figure 6A shows the identification of two GS polypeptides, GS2 (45 kDa) and GS1 (40 kDa) in the leaf lamina. The GS1 polypeptide was predominant in stems and roots.

In order to investigate the correspondence of *GS *trancripts and GS polypeptides in the different organs, total proteins from the same protein samples (leaves, stems and roots) were also separated by two-dimensional gel electrophoresis (2D-PAGE), and the GS polypeptides identified by western blotting (Figure 6B). This experimental approach allowed us to identify GS polypeptides of different charge among the family of GS polypeptides of the same size. Thus, in the leaf lamina the GS2 polypeptide was resolved as several spots with the most abundant exhibiting a calculated isoelectric point (pI) of 5.26. The GS1 polypeptide was resolved as a major spot of a pI of 5.52. In the stem, two major major spots corresponded to GS1 polypeptides of pI 5.20 and 5.81. Finally, in the roots the major GS1 spot had a calculated pI of 5.14. These experimental pI values were in the range of the predicted pI values for poplar GS polypeptides (Table 1).

### Seasonal changes in GS gene expression

**Figure 7:**
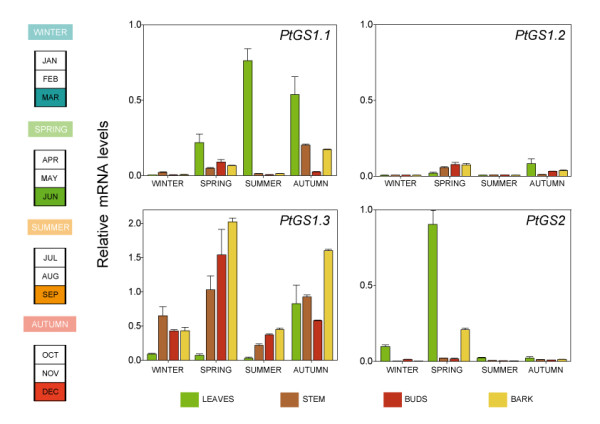
**Seasonal changes of *GS *gene expression in poplar trees**. Total RNA was extracted from leaves, stem, buds and bark of 10-year-old hybrid poplar trees. Transcript levels of *PtGS1.1*, *PtGS1.2*, *PtGS1.3 *and *PtGS2 *were determined by real-time qPCR analysis as described. Expression levels are presented as relative values to reference genes (actin2 and ubiquitin). The histograms represent the mean values of at least three independent experiments with standard deviations.

We were also interested to know the seasonal changes in the expression of the *GS *gene family in poplar. Transcript levels of *PtGS1.1*, *PtGS1.2*, *PtGS1.3 *and *PtGS2 *were quantitatively determined in RNA extracts from leaves, stem, buds and bark of 10-year-old poplar trees (*Populus tremula *x *P. alba*, clone INRA 7171 1-B-4). Figure 7 shows that *GS *duplicates exhibited contrasting patterns of gene expression during annual growth. The expression of the *PtGS1.1 *duplicate was very low during winter and increased during spring to reach the maximum values at the end of summer and autumn. Interestingly, the peak values of transcripts were observed in leaves. Transcript abundance for the *PtGS1.2 *duplicate was low in all samples examined at the different seasons of the year. *PtGS1.3 *was highly expressed in stems buds and bark during all seasons with peak transcript levels during spring and autumn. Interestingly, the levels of *PtGS1.3 *transcripts were low in leaves except in autumn when levels increased significantly. Finally, high levels of *PtGS2 *transcripts were exclusively detected in expanding leaves in spring.

## Discussion

The *GS *gene family in poplar consists of 8 different genes which exhibit all structural and regulatory elements to be potentially considered as functional genes (Table 1). A detailed analysis of the genomic *GS *sequences suggests that the *GS *gene family in poplar is organized into 4 groups of duplicated genes, *PtGS1.1*, *PtGS1.2*, *PtGS1.3 *and *PtGS2*. These *GS *genes are distributed on separate loci in different chromosomes, and to our knowledge, *Populus trichocarpa *is the first plant species in which the complete *GS *family is observed to be duplicated. However, the duplication of a single *GS *gene has been previously reported in plants. Thus, two copies of *GS1 *genes have been described in *Pisum sativum *[20], and more recently the occurrence of two distinct *GS2 *genes have been reported in *Medicago truncatula *[21]. Homology-microsynteny analysis of the genomic regions where the *GS *genes are located strongly suggests that the origin of the duplicated genes is a genome-wide duplication event that occurred approximately 65 Myr and which is still detectable over approximately 92% of the poplar genome [17]. Following duplication, new copies of a gene may undergo modifications allowing functional diversification, which is a significant source of evolutionary novelty in plants [22]. However, it is also possible that a duplicated gene copy is rapidly lost through pseudogenisation. Interestingly, the exon-intron organization is highly conserved in each pair of duplicated genes in poplar and similar regulatory elements are present in their promoters. These findings provide evidence supporting the expression of *GS *duplicates in the same cell-types where they are subjected to similar developmental and environmental cues. Furthermore, their coding regions are also quite well-conserved, indicating they encode for essentially the same or very similar GS enzymes. All these results suggest that these duplicated genes could play equivalent roles in poplar nitrogen metabolism.

The molecular and functional analyses of *GS *gene families in other plants revealed specialization of GS isoenzymes to fulfil specific and non-overlapping roles in nitrogen metabolism depending of the tissue and plant species [9,10]. Phylogenetic analyses of poplar *GS *genes have shown that genes encoding chloroplastic and cytosolic isoforms form two sister groups as previously described for other *GS *gene families [10]. It has been suggested that the two groups of genes (*GS1 *and *GS2*) diverged by duplication from a common ancestor [23] and that this separation occurred before the divergence of gymnosperms/angiosperms [5] but possibly after the appearance of vascular plants [24]. It has been proposed that the gain of a N-terminal transit peptide in GS2 would provide adaptive advantages to plants through enhanced photorespiratory ammonium assimilation in the plastids [12]. Members of the GS1 clade in angiosperms are grouped in subfamilies as previously reported by others [6,10,21]. *PtGS1.2 *and *PtGS1.3 *duplicates were found associated to these subfamilies suggesting they could play similar functions to those described for these isoforms. In contrast, the *PtGS1.1 *duplicate was found separated from *PtGS1.2 *and *PtGS1.3 *genes.

The intron-exon organization of the poplar *GS *genes supports the above hypothesis (Figure 3). The positions and lengths of exons are quite similar for all genes suggesting that the structure of the ancestral *GS *gene has been maintained during evolution with some modifications, such as the presence of a plastid targeting sequence in the first exon of *GS2 *and minor changes in some other exons of *GS1 *genes.

A detailed analysis of *GS *transcript abundance in different tissues and organs of poplar allowed us to identify specific expression patterns of the individual members of the gene family (Figures 5 and 7). *PtGS2 *transcripts were most abundant in leaves as previously reported for other angiosperms where the GS2 isoform is responsible for assimilation of photorespiratory ammonium [9,10]. In fact, the promoters of the poplar *GS2 *duplicates contained *cis *regulatory elements described in other *GS2 *genes in angiosperms [18]. An additional role of GS2 is the assimilation of nitrate-derived ammonium in leaves. It is well known that plants differ in the localization of nitrate reduction and assimilation. Thus, some species localize nitrate reduction and assimilation in the roots, whereas other species assimilate nitrate preferentially in the leaves. In poplar, most nitrate assimilation takes place in the leaves [25]. Therefore, high levels of the GS2 isoform are necessary to assimilate the ammonium generated by nitrate reduction within the chloroplast.

Only one of the three *PtGS1 *duplicates in poplar, *PtGS1.1*, was also preferentially expressed in leaves and interestingly its expression pattern spatially complemented the observed expression pattern of *PtGS2*. Thus, *PtGS1.1 *transcripts were particularly abundant in the older leaves located at the basal part of the tree. These results suggest a relevant role of *PtGS1.1 *in glutamine biosynthesis associated to photosynthetic metabolism in leaves. Furthermore, the presence of light-regulation boxes [26,27] in the promoter regions of *PtGS1.1 *duplicates (Figure 4) is consistent with our data and may explain the above described expression pattern in green leaves.

Poplar *GS1.2 *was preferentially expressed in roots of young trees suggesting a role for this gene duplicate in primary assimilation of nitrogen from soil, as it has been previously described for other cytosolic GS enzymes in plants [28-30]. Interestingly, the relative abundance of *PtGS1.2 *transcripts increased significantly (12 fold) in poplar leaves infected with *Pseudomonas syringae*, whereas the expression of other members of the *GS *gene family was not affected (data not shown). The induction of a GS1 gene in response to pathogen attack has been previously described [31,32]. Moreover, it has been demonstrated in infected tomato leaves and senescing tobacco leaves that the cytosolic isoform involved in nitrogen remobilisation is the product of a *GS1 *gene preferentially expressed in roots [33,34]. These data, together with our work described here suggest that *PtGS1.2 *may have a role in nitrogen remobilization during leaf senescence.

In young trees, the maximum expression levels of the twin *PtGS1.3 *genes were detected in stems and petioles. Furthermore, this member of the poplar *GS *family exhibited the highest levels of gene expression suggesting it plays an essential role in nitrogen metabolism. The regulatory regions of the *PtGS1.3 *duplicates contained AC elements involved in the interaction with members of the R2R3 Myb factors regulating the transcription of genes for lignin biosynthesis [35,36]. Similar cis-regulatory elements and trans-acting factors have been found to coordinate lignin biosynthesis and nitrogen recycling in pine [37], suggesting that *PtGS1.3 *is involved in nitrogen recycling associated to lignification in poplar. Transcriptomic analyses have also suggested a role of Dof family members in the regulation of genes under conditions resulting in increased lignin deposition [38]. The differential regulation of cytosolic GS genes in conifers by a member of the Dof family (Dof5) was recently reported [39] and putative regulatory elements for Dof regulation have been identified in poplar *GS *genes (Figure 4). Furthermore, we have found that orthologous Dof factors are also involved in the regulation of GS isoforms in poplar (García-Gutiérrez, Avila C, Cánovas FM, unpublished data). The analysis of GS polypeptides in different poplar organs by 2D-PAGE (Figure 6) largely confirmed the expression patterns determined for the duplicated *GS *genes. The GS polypeptides were resolved in four major spots with differential accumulation in poplar organs. Thus, in the leaves, the GS2 and GS1 polypeptides displayed pI values in the range of the calculated pI values for the *PtGS2 *and *PtGS1.1 *gene expression products (Table 1). In stems, the predominant GS1 polypeptide is predicted to be the expression product of the *PtGS1.3 *duplicate whereas the major GS1 polypeptide in roots is predicted to be the expression product of *PtGS1.2*. This conclusion is supported by the close similarity between the pI values of the GS1 isoforms separated in Figure 6 and the corresponding values deduced from the polypeptides encoded by the *PtGS1.3 *and *PtGS1.2 *duplicates (Table 1).

The analysis of transcripts in adult trees during one year of growth (Figure 7) showed that the expression of the poplar GS family members is seasonally regulated. The expression of the *PtGS2 *duplicate was high in leaves in spring when photosynthesis and photorespiration are at maximum levels [40]. Furthermore, glutamine is required to initiate vegetative protein accumulation during new shoot development in spring [41]. Developing leaves represent a strong sink for nitrogen during active growth [42]. High levels of *PtGS1.1 *gene expression were also found in leaves of adult trees in summer and autumn when the expression of *PtGS2 *is very low. These data suggest that the GS1.1 isoform could play an important role in the redistribution of nitrogen from poplar leaves to stems during summer and autumn. In leaves, stem, buds and bark of adult trees, extremely low levels of *PtGS1.2 *transcripts were detected in winter and summer, however, slight increases were observed in spring and autumn. Nitrogen mobilisation in poplar is seasonally regulated with recycling and transport of nitrogen compounds from senescing tissues to storage tissues in autumn and remobilisation of nitrogen reserves to support active growth in spring [42]. In adult trees, *PtGS1.3 *gene expression was seasonally regulated with particularly high transcript levels in spring and autumn. Transcript abundance was much high in heterotrophic tissues such as stem, buds and bark, with the exception of senescent leaves in autumn.

The role of cytosolic GS in poplar growth and biomasss production has been reported previously [13,14]. Furthermore, enhanced *GS *expression in poplar results in enhanced efficiency of nitrogen assimilation [16]. The role of GS1 in ammonium assimilation and nitrogen remobilisation is particularly important in perennial plants that are able to cope with recurrent periods of growth and dormancy. For example, trees divert large amounts of carbon to the biosynthesis of phenylpropanoids needed to generate lignin, an important constituent of wood. Although lignin does not contain nitrogen, during wood formation there is significant release of nitrogen in the form of ammonium when phenylalanine is deaminated and channeled into lignin biosynthesis and when glycine is decarboxylated in C1 metabolism. These two metabolic pathways are active in lignifying cells [43]. Ammonium ions released must be reintegrated into metabolism in order to maintain high rates of lignification without affecting nitrogen economy [12,44]. In fact, poplar GS1 transcripts and polypeptides accumulate in developing xylem cells where activities of enzymes involved in the phenylpropanoid pathway and C1 metabolism are high and, therefore, ammonium is liberated [45,46]. According to these findings we decided to examine in silico the expression of GS genes during wood formation in hybrid poplar (*Populus tremula *x *Populus tremuloides*) using the microarray data available in *Populus *DB [47]. Principal component analysis (Additional file 1) showed a high degree of co-expression for *PtGS1.3 *and relevant genes involved in lignin biosynthesis and C1 metabolism. In contrast, other members of the *GS *family also expressed during poplar wood formation (*PtGS1.1 *and *PtGS2*) did not show such correlation. Taken together, these data suggest an essential role of *PtGS1.3 *in lignifying tissues of poplar.

## Conclusion

**Figure 8:**
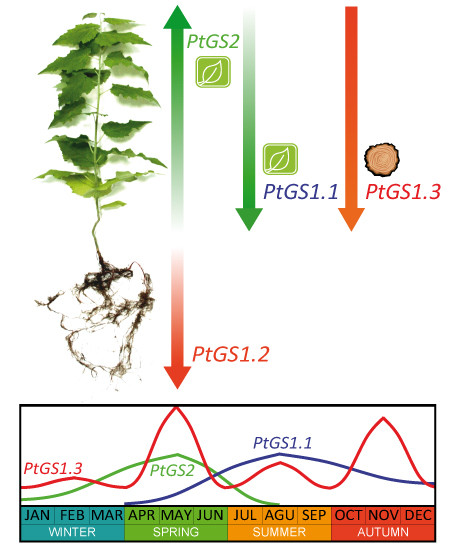
**Schematic representation of the spatial and seasonal regulation of the poplar *GS *family**. The arrows and intensity of colors indicate the existence of a gradient of gene expression along the plant axis. Maximum values of gene expression in leaves, stems and roots were considered in the scheme. Global seasonal variation reflects major changes in gene expression.

In the present study the structural and expression analysis of the *GS *gene family in poplar is presented. The *GS *gene family consists of 8 different genes exhibiting all structural and regulatory elements consistent with their roles as functional genes. Our results indicate that the family members are organized in 4 groups of duplicated genes, 3 of which code for cytosolic GS isoforms (GS1) and 1 codes for the chloroplast located GS isoform (GS2). Detailed expression analyses have revealed specific spatial and seasonal patterns of *GS *gene regulation in poplar (Figure 8). These data provide insights into the metabolic function of GS isoforms in poplar and pave the way for future functional studies.

Our analysis shows that *Populus trichocarpa *is the first plant species in which it was observed the complete *GS *family duplicated. Considering all data in the present paper, it appears that *GS *genes have been highly conserved following whole-genome duplication in poplar. In contrast, duplicated members of other gene families in poplar have rapidly diverged [48,49]. Some authors argue that genes involved in transcriptional regulation and developmental genes are preferentially retained [50]. It has also been proposed that one of the factors influencing the probability of duplicate gene retention is its connectivity [22]. Our data suggest that *GS *duplicates could have been retained in order to increase the amount of enzyme in a particular cell type. This possibility could contribute to the homeostasis of nitrogen metabolism in functions associated to changes in glutamine-derived metabolic products. It has been reported that recently duplicated genes play an important role in the functional compensation of metabolic products in *Arabidopsis *[51]. The presence of duplicated *GS *genes in poplar could also contribute to diversification of the enzymatic properties for a particular GS isoform through the assembly of GS polypeptides into homo oligomeric and/or hetero oligomeric holoenzymes in specific cell types. Further research is needed to confirm these hypotheses.

## Methods

### Plant material

Experiments were performed with hybrid poplar (*Populus tremula *x *Populus alba*, clone INRA 717 1-B4, Institut National de la Recherche Agronomique, INRA). For the expression analysis in different plant organs, plants were micropropagated in vitro on half-strength Murashige and Skoog medium (MS) as described previously [13]. Unless otherwise noted, plantlets were maintained under conditions described previously [13,14]. Rooted shoots were transferred to plant growth chambers in plastic pots containing a potting mix (HM3-Agromálaga, Málaga, Spain) and vermiculite in proportions 1:1. After ex-vitro acclimatization, the plantlets were maintained for 8 weeks in the following conditions: temperature was kept constant at 22°C, day-length was set at 16 hours, light was supplied at an intensity of 125 μmol m^2 ^s^-1 ^and humidity was fixed about 80%. Plants were regularly supplied with a nutrient solution containing 10 mM potassium nitrate. At the end of the growing period, *P. tremula *x *P. alba *plants were harvested and samples taken from 15 different sections from the shoot apex to the root tip.

The aerial regions of the plants was divided into four parts: the apical bud (A), the 1st, 2nd, 3rd, 4th and 5th apical leaves (A1); the intermediate region with the 6th, 7th, 8th, 9th and 10th leaves (A2), and the more basal region including 11th, 12th, 13th, 14th and 15th leaves (A3). Each section was further divided in: L, leaf lamina; V, principal midrib; P, petiole; S, stem. The root was sectioned in R1, principal root and R2, secondary root.

Seasonal analysis of gene expression was monitored in 10-year-old trees of hybrid poplars located in the experimental centre Grice-Hutchinson, Barrio de San Julián, 29004, Málaga, Spain. The samples used were leaves, stems, buds and bark from hybrid trees. The samples were harvested in mid- March, June, September and December of 2008.

### Identification of GS gene duplicates and chromosomal mapping

The *GS *nucleotide and protein sequences were identified using the Eukaryotic Orthologous Groups section of the Join Genome institute (http://genome.jgi-psf.org/Poptr1_1/Poptr1_1.home.html). The protein identifiers used in this genome portal have been conserved in our study (Table 1). GS sequences captured from the poplar database were annotated by blastp searches in the GenBank (http://blast.ncbi.nlm.nih.gov/Blast.cgi). The p-distances among the exonic and intronic regions of the GS family were calculated with the phylogenetic program MEGA 4 [52], sewing the intron sections of each gene in continuous sequence. The degree of identity between the genomic regions in which the *GS *duplicated genes were located was explored by using a strategy of searching microsynteny among the different Linkage Groups. Upstream and downstream genomic sequences were aligned with CLUSTALW [53] and the ORF sequences flanking the *GS *genes were located manually. Results were then compared with those reported by [17].

### Sequence and phylogenetic analysis

The alignment of the *Populus trichocarpa *GS protein sequences was carried out with CLUSTALW [53]. The protein sequences used in the construction of the phylogenetic tree were collected from species whose genomes have been fully sequenced. Additionally, sequences of other interesting arboreal species, including *Pinus *or *Picea*, have been included. The protein sequences, and their corresponding identifiers, were found in the following databases:

*Populus trichocarpa *and *Sorghum bicolor*: http://genome.jgi-psf.org/

*Pinus sylvestris *and *Picea sitchensis*: http://www.ncbi.nlm.nih.gov/Genbank/index.html.

*Arabidopsis thaliana*: http://www.arabidopsis.org/

*Oryza sativa*: http://rice.plantbiology.msu.edu/

*Vitis vinifera*: http://www.phytozome.net/

The phylogenetic tree was constructed using the phylogeny platform http://www.phylogeny.fr/[54] and comprised the following steps. Sequences were aligned using T-Coffee (v6.85) using the 10 best local alignments (Lalign_pair), an accurate global alignment (slow_pair). After alignment, ambiguous regions were removed with Gblocks (v0.91b). Minimum length of a block after gap cleaning was set at 10; no gap positions were allowed in the final alignment. All segments with contiguous non-conserved positions greater than 8 were rejected. The minimum number of sequences for a flank position was set at 85%. The phylogenetic tree was reconstructed using the neighbor joining method implemented in the BioNJ program, using as substitution model a Dayhoof PAM matrix and including 1000 bootstraps. Distances were calculated using ProtDist. The DAY substitution model was selected for the analysis [55].

### Promoter analysis

The presence of regulatory elements in the 5'-upstream region of poplar *GS *genes was analyzed starting in the ATG codon of initiation of translation. Sequences for each pair of duplicated genes were aligned by means of MultAlin [56] in order to locate common regions, and those showing high identity were analyzed to identify putative cis regulatory elements in the plant databases PLACE [57] and PlantCARE [58]. Sequence stretches of 600 base pairs for the GS1 genes and 1300 base pairs for GS2 genes were compared. More distant regions were not considered, because the p-distance values in these regions showed that similarity between the duplicated sequences diminished considerably.

### RNA extraction and cDNA synthesis

Samples from photosynthetic tissues (0.25 g) or non-photosynthetic tissues (0.5 g) were ground under liquid nitrogen with a mortar and pestle in proportions 1:2 and 1:1, respectively. RNA extraction was performed as described by Canales et al. [59] with minor modifications. The RNA was quantified with a NanoDrop ^® ^ND-1000 spectrophotometer (NanoDrop Technologies, Inc. Wilmington, USA). The integrity of RNA in the samples was verified by agarose gel electrophoresis.

The cDNA synthesis was performed by means of the PrimeScriptTM RT reagent (Perfect Real Time) of Takara BIO Inc. (Otsu, Shiga Japan), following the instructions recommended by the manufacturer. The reaction mix contained 0.5 μg of total RNA in a final volume of 10 μL, which were incubated during 15 min at 37 °C.

### Real-time quantitative PCR (qPCR)

The 3' untranslated regions of *PtGS1.1*, *PtGS1.2*, *PtGS1.3 *and *PtGS2 *duplicates were compared to identify sequences with high identity for each pair of duplicated genes. Oligonucleotide primers were designed to amplify specifically the transcripts encoded by each pair of duplicated genes. Sequences of forward and reverse primers follow.

*PtGS2*-F: GGAGCATCACTTGGATCTAGATGG

*PtGS2*-R: CAAAACCCAAGAGTAAAAAGGTCC

*PtGS1.1*-F: ATGGTTGTCTGTCAATTTGTTTGCC

*PtGS1.1*-R: CCAGCAAGAGTTTTATTTAGATTAG

*PtGS1.2*-F: GGAATTGAGTATTGGAAGATGATGG

*PtGS1.2*-R: TATGTTCATAAATGATCAACAGCC

*PtGS1.3*-F: TGGAAACCATAAGAGATCACCACC

*PtGS1.3*-R: GAAGAGGCAATTCTTGTACCAAG

PCR products were verified by melting point analysis at the end of each experiment. The identity of the PCR products for each *GS *duplicate was tested during protocol development by gel electrophoresis and confirmed by DNA sequencing (Additional file 2).

The relative quantification of the gene expression was carried out by qPCR using a thermal cycler (Real System Stratagene MX Swindles PCR 3000PTM, Agilent Technologies, Santa Clara, CA). The qPCR system was the QUANTIMIX EASY SYG (Biotools B&M Labs S.A. Madrid, Spain) and the protocols followed were those recommended by the manufacturer. The PCR reactions were performed by triplicate using samples without DNA as controls. The volume of the qPCR samples was 2 μL containing 10 ng of cDNA from the RT reactions. The amplification program had 3 steps: i) 1 cycle (95°C, 2 min); ii) 40 cycles, cDNA denaturing (95°C, 15 s), hybridization (55°C, 15 s) and extension (72°C, 30 s); iii) 1 cycle (95 °C, 1 min) and 1 cycle (30 s) for absolute temperatures from 55°C to 95°C to generate the dissociation curve in order to confirm the specific amplification of each individual reaction.

The relative expression levels were calculated by using actin2 and ubiquitin as reference genes [60]. The initial number of transcripts of the candidate and reference genes (N_0_) was calculated by means of the LinRegPCR software version 11.0 [61]. The normalized N_0 _was found by calculating the ratio between the averages of the N_0 _of the replicates and the N_0 _of the reference genes (normalization factor).

### Protein extraction and western blot analysis

Poplar protein extraction was performed using the following protocol. One gram of plant tissue was homogenized in mortar and pestle with 1 g of sand and 1 mL of extraction buffer [0.175 M Tris pH 8.8, 5% SDS (w/v), 15% glycerol (v/v), 0.3 M mercaptoethanol]. The extract was then centrifuged at 10,000 × g, 4°C for 30 min. The supernatant was mixed with 4 volumes of acetone for 1 h at -20°C and then centrifuged at 10,000 × g 4°C for 30 min. The resulting pellet was washed with 80% (v/v) acetone and centrifuged two times at 10,000 × g 4°C for15 min. The pellet was dried and solubilized in loading buffer for SDS-electrophoresis on 12.5% (w/v) polyacrylamide gels. Resolved polypeptides were electrotransferred onto nitrocellulose membranes (Whatman GmbH, Dassel, Germany) and the presence of GS polypeptides was immunorevealed as described by Cánovas et al. [62] using the antiserum raised against recombinant pine GS [19]. Subsequent detection of immunocomplexes was carried out by a peroxidase assay.

### Two-dimensional gel electrophoresis

Two-dimensional gel electrophoresis was carried out as described previously [63]. Poplar proteins were solubilized in isoelectrofocusing (IEF) loading buffer. The amount of protein loaded per gel was 30 μg. IEF slab gels were 80 × 70 × 1 mm consisting of 5% (w/v) polyacrylamide, 8.3 M urea, 2.5% (v/v) carrier ampholytes (Pharmalyte pH 4-6). IEF was performed at 200 V for 2.5 h. The second dimension electrophoresis was also performed in 80 × 70 × 1.5 mm slab gels of 12.5% (w/v) polyacrylamide gels containing SDS as described above. The resolved proteins were then transferred to nitrocellulose membranes and immunodetection was performed essentially as described for one-dimensional western blots.

### Protein quantification

Protein levels were determined by the Bradford's procedure [64]. In samples solubilized with SDS protein contents were estimated as described by Ekramoddoullah [65].

## Authors' contributions

VCR carried out experiments. AGG contributed bioinformatic analyses and did illustrations. JC contributed the qPCR work. CA contributed protein work. AGG, CA and FMC conceived this study. EGK and FMC wrote the manuscript. All authors read and approved the final manuscript.

## Supplementary Material

Additional file 1 **Principal Component Analysis (PCA) of *GS *genes and genes involved in lignin biosynthesis and C1 metabolism**. The expression profiles of *GS *genes were examined in silico during wood formation in hybrid poplar (*Populus tremula *x *Populus tremuloides*) using the microarray data available in *Populus *DB [48]. Tissue samples were collected from five positions to cover xylem development from cambium meristematic cells: cambium, early expansion, late expansion, secondary wall formation and late cell maturation. *GS *genes: *PtGS1.1-710678*, *PtGS1.3-827781 *and *PtGS2-725763*; Lignin genes: *PtPAL-696959*, *PtCCoAMT-691730*, *PtAldOMT-345776*; C1 metabolism genes: *PtGDC-H-570626*, *PtMTHFR-654300*, *PtAHCY-540785*, *PtMTR-738504 *and *PtMAT-689393*. Plot of the analyzed variables (gene expression levels during lignification) on the two first principal components: 90.8% y 3.3% of the variance respectively. Most of the gene co-expression values were positively correlated with the first principal component. The second principal component is mainly characterized by the mutually exclusive expression of *PtGS1.1-710678 *and *PtGS2-725763 *respectively. Click here for file

Additional file 2 **Predicted and sequenced cDNAs of poplar *GS *genes**. Predicted and sequenced cDNAs of poplar *GS *genes are listed. Click here for file
