# Supplementary figures and images for: The glutamine synthetase gene family in Populus
Source: BMC Plant Biol. 2011 Aug 25;11:119. doi: 10.1186/1471-2229-11-119 (PMC3224142; doi:10.1186/1471-2229-11-119)

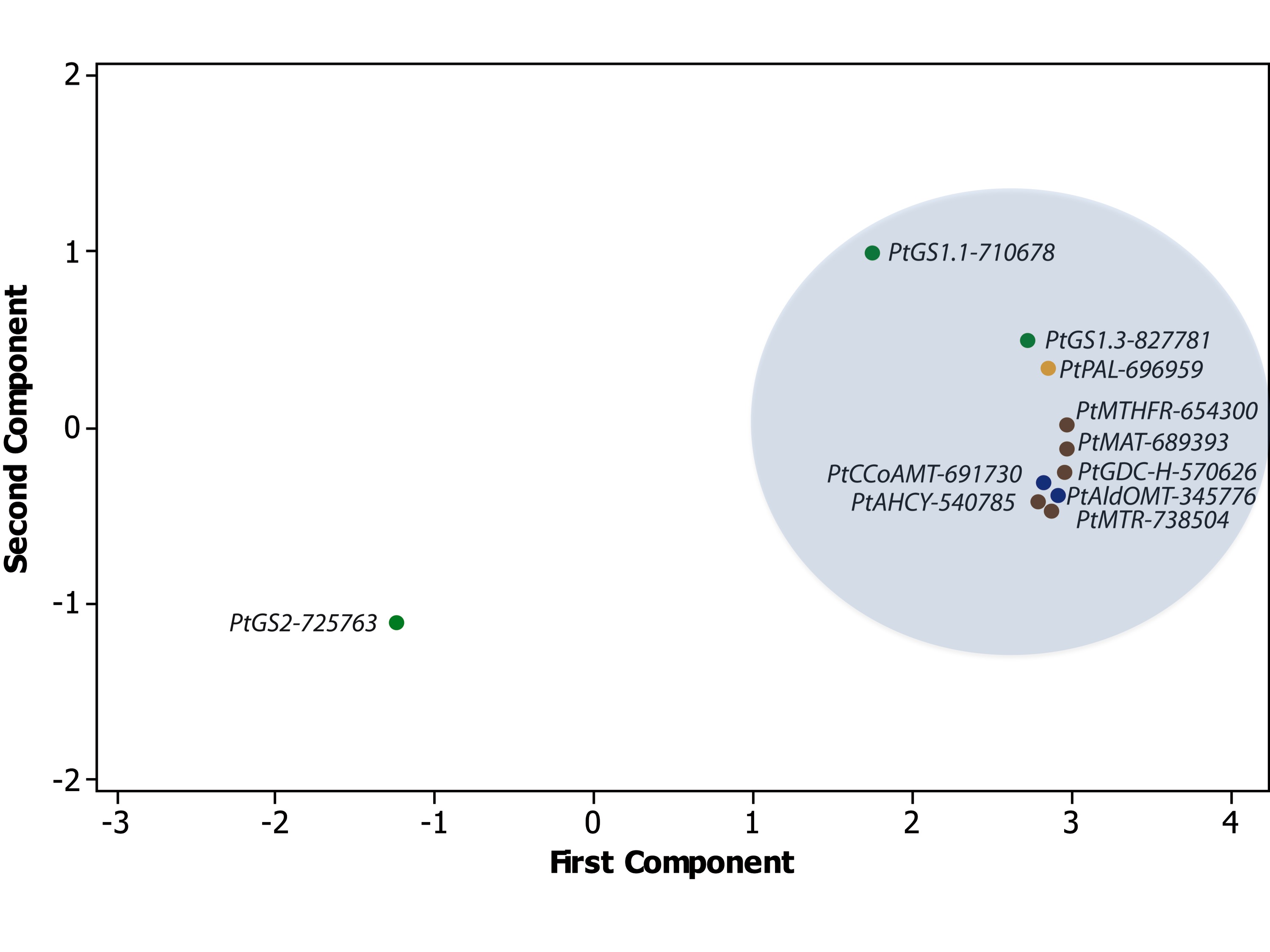

Supplement: Additional file 1 — Principal Component Analysis (PCA) of GS genes and genes involved in lignin biosynthesis and C1 metabolism. The expression profiles of GS genes were examined in silico during wood formation in hybrid poplar (Populus tremula x Populus tremuloides) using the microarray data available in Populus DB [48]. Tissue samples were collected from five positions to cover xylem development from cambium meristematic cells: cambium, early expansion, late expansion, secondary wall formation and late cell maturation. GS genes: PtGS1.1-710678, PtGS1.3-827781 and PtGS2-725763; Lignin genes: PtPAL-696959, PtCCoAMT-691730, PtAldOMT-345776; C1 metabolism genes: PtGDC-H-570626, PtMTHFR-654300, PtAHCY-540785, PtMTR-738504 and PtMAT-689393. Plot of the analyzed variables (gene expression levels during lignification) on the two first principal components: 90.8% y 3.3% of the variance respectively. Most of the gene co-expression values were positively correlated with the first principal component. The second principal component is mainly characterized by the mutually exclusive expression of PtGS1.1-710678 and PtGS2-725763 respectively. [file 1471-2229-11-119-S1.JPEG]
